# Fatal anaphylaxis due to peanut exposure from oral intercourse

**DOI:** 10.1186/s13223-021-00611-9

**Published:** 2021-10-18

**Authors:** Lundy R. McKibbin, Sidney Kin-Hung Siu, Hannah T. Roberts, Michael Shkrum, Samira Jeimy

**Affiliations:** 1 Division of Clinical Immunology and Allergy, Department of Medicine, St. Joseph’s Hospital, Western University, 268 Grosvenor Street, London, ON N6A 4V2 Canada; 2 Department of Pathology and Laboratory Medicine, University Hospital and Schulich School of Medicine and Dentistry, Western University, London, ON Canada

**Keywords:** Anaphylaxis, Asthma, Peanut allergy, Food allergy, Mortality

## Abstract

**Background:**

Intimacy-related allergic reactions, including anaphylaxis, are under-reported due to social stigma, lack of awareness, and misdiagnosis. The differential diagnosis for intimacy-related anaphylaxis is extensive and includes systemic human seminal plasma allergy, exercise-induced anaphylaxis, asthma exacerbation, latex allergy, and transference of food or drug allergens through saliva or seminal fluid.

**Case presentation:**

Two adolescents met on a popular dating phone application. One individual had a long-standing history of asthma and peanut allergy. Although they never kissed, the male with peanut allergy received fellatio, while the other male had eaten peanut butter before they met. During fellatio, the peanut allergic male developed respiratory symptoms, used his bronchodilator, and collapsed. He remained unconscious despite aggressive interventions by emergency personnel called to the site. The clinical history and autopsy results suggested anaphylaxis to peanut allergen exposure from the intimate exposure as the cause of death.

**Discussion and conclusions:**

To date, nearly all reported cases of intimacy-related anaphylaxis involve symptomatic women. This is the first report of intimacy-related anaphylaxis involving men who have sex with men and the first report of potential allergen transfer from oral mucosa to a patient receiving fellatio. Based on the paucity of published cases, death from intimacy-related anaphylaxis is exceedingly rare. Post-mortem analysis is inherently difficult, as an elevated tryptase level has myriad potential causes; nevertheless, the authors suggest that intimacy-related anaphylaxis due to peanut allergy is the most likely diagnosis. With increasing popularity of relationship applications, especially amongst stigmatized populations, this case highlights the importance of allergy awareness and patient education to decrease risk, particularly in the adolescent population, who are already at increased risk of severe anaphylaxis. Especially amongst those participating in intimate activities, disclosure of one’s allergies warrants discussion, as the outcome can be fatal. Our case demonstrates the crucial need for increased advocacy in food allergy, education around intimacy-related anaphylaxis, and the importance of allergy awareness and prevention across all populations.

## Background

The differential diagnosis for intimacy-associated anaphylaxis is broad, and includes latex hypersensitivity, systemic human seminal plasma allergy, transference of food and drug allergens in seminal fluid, post orgasmic illness syndrome, exercise-induced anaphylaxis, and sexercise-induced asthma [1, 2].

Adverse reactions to intimate activity like kissing can be life threatening, and as little as 50 µg of peanut can be sufficient to induce allergic symptoms [1, 2]. Symptoms may occur up to 6 h after peanut ingestion; in one report, anaphylaxis occurred 2 h after ingestion despite brushing the teeth, rinsing the mouth, and chewing gum [2]. Salivary analyses have shown that one of the major allergenic peanut proteins, Ara h 1, can persist in up to 10% of individuals several hours after ingestion [1, 2]. Given the persistence of peanut allergen in the oral cavity, and high solubility of peanut, intact skin and mucosa can be penetrated, making other types of intimate contact important risk factors for allergic reactions and anaphylaxis [1, 2].

## Case presentation

We present the first case report of intimacy-related fatal anaphylaxis due to accidental food allergen transfer, after two adolescent males met via a dating application and engaged in oral intercourse. One male ingested peanut butter before the encounter, unbeknownst to the other male, who had an IgE-mediated peanut allergy, allergic rhinitis, and a history of severe asthma. During insertive fellatio, he developed acute wheeze and dyspnea. He did not have urticaria, angioedema, or gastrointestinal symptoms. He used his short-acting bronchodilator and collapsed shortly after developing respiratory symptoms and a personal epinephrine autoinjector was not administered. Forty-five minutes later, emergency medical services personnel arrived and intubated the patient. Palpation and electrocardiogram demonstrated pulseless electrical activity. He received cardiopulmonary resuscitation and multiple doses of intravenous epinephrine. He was then started on infusions of norepinephrine, epinephrine, and salbutamol after return of spontaneous circulation, and was admitted to the local hospital. He received pulse-dosed corticosteroids and bicarbonate. Death was pronounced the next day. The immediate cause of death was pneumonia due to hypoxic ischemic encephalopathy, as a result of cardiorespiratory arrest from anaphylaxis.

The male allergic to peanut was well prior to the encounter and his asthma control had improved on regular medical therapy with clinical control achieved, as per Canadian Thoracic Society criteria, corroborated by improved pulmonary function test parameters at his most recent allergy assessment (Table 1). The patient was followed by Allergy and Respirology. He had avoided peanut and carried an epinephrine autoinjector since he was a toddler for IgE-mediated peanut allergy, initially diagnosed due to immediate urticaria after consumption with an associated positive skin test. Two months before his death, clinical assessment and skin prick testing confirmed persistent peanut allergy with a strongly positive skin test to peanut.

**Table 1:** Clinical and Demographic Patient Characteristics

Test	Data collected	Results
Skin prick testing (Food Allergy)	2 months prior to death	Peanut: 12 mm Tree nuts: negative
Skin prick testing (Environmental aeroallergens)	1.5 years prior to death	Positive to tree and grass pollens, cat, dog, molds
Pulmonary function testing	2 weeks prior to death	FEV_1_ 3.65 L (82% predicted), FVC 4.70 L (91% predicted), FEV_1_/FVC 0.78, and no significant post bronchodilator response^a^
Total IgE	2 weeks prior to death	500 units/milliliter (normal range 0–400)
Tryptase	Serum sample-Emergency Department	47.0 µg/L
Medications		1. Budesonide/Formoterol 200/6 mcg 2 inhalations twice daily as maintenance, with additional inhalations for rescue, up to 8 inhalations maximum per day 2. Tiotropium (Spiriva Respimat) 2.5 mcg two inhalations once daily 3. Salbutamol 100 mcg MDI prn 4. Montelukast 10 mg daily 5. Rupatadine 10 mg daily prn

^a^Salbutamol administered 1.5 h before the appointment. MDI, metered dose inhaler; SPT, skin prick test

## Discussion and conclusions

This is the only reported case of a patient developing anaphylaxis after insertive, rather than receptive, fellatio [1]. Published cases of anaphylaxis after fellatio have exclusively involved the individual performing the act—receptive fellatio—and never a male engaged in insertive fellatio [1, 2]. Most cases involve a reaction to seminal fluid or allergen contents temporarily present in the seminal fluid. In two cases, male partners developed allergic symptoms after penetrative intercourse; supporting the notion that allergens can penetrate the mucous membrane of the glans penis [3, 4]. Our case is novel in that the individuals never kissed, demonstrating the ability of peanut allergen to penetrate other mucous membranes, resulting in severe anaphylaxis.

The patient’s tryptase level was significantly elevated at 47.0 µg/L (normal reference range 3.8–11.4 µg/L) in keeping with a diagnosis of anaphylaxis in a living patient. The sample was drawn in the emergency department, after the patient was in cardiac arrest for at least 45 min. Whether this interval influenced the tryptase level is uncertain*. *In vivo tryptase level cut offs do not apply to post-mortem results, which must be interpreted in the context of clinical and autopsy findings [5]. Regardless of whether the tryptase level in this case is considered an antemortem or a post-mortem result, various studies have shown that the concentration seen in this case was in the range found in anaphylactic deaths. A post-mortem analysis of 20 deaths due to anaphylaxis determined that the optimal cut-off tryptase level, determined from femoral vein samples, was 43 µg/L (98% specificity; 90% sensitivity) [5]. Sun et al. in a systematic review of nine reports found that a tryptase level of 30.4 µg/L distinguished anaphylactic from non-anaphylactic deaths [6]*.* Tejedor-Alonso et al. found that the optimal cut-off tryptase concentration distinguishing anaphylaxis from non-anaphylaxis cases was 64 µg/L (95.5% specificity; 74.4% sensitivity); however, anaphylaxis from food allergy is known to result in lower tryptase levels than anaphylaxis from other causes [7]. These studies further support our case as consistent with anaphylaxis due to peanut exposure from oral intercourse.

Several other factors support anaphylaxis as the cause of death in this patient, in addition to the elevated tryptase level. The patient had respiratory compromise and cardiovascular collapse after mucosal exposure to a known food allergen, in keeping with a diagnosis of anaphylaxis. A personal epinephrine autoinjector was not administered during the event. Toxicological analysis did not find any evidence of drugs of abuse. Condoms were not used, making other causes of anaphylaxis, such as latex anaphylaxis, unlikely. An additional diagnostic consideration for this case is postcoital asthma, termed sexercise-induced asthma or honeymoon asthma [8]. Parasympathetic and cholinergic over activity in addition to heightened emotions are thought to provoke severe, life-threatening symptoms invoked by sexual arousal, but not by exercise [8]. Symptoms may be pre-coital or occur up to 6 h after coitus and have even required mechanical ventilation [8]. Our patient achieved clinical control of his asthma, as per Canadian Thoracic Society criteria, corroborated by improved pulmonary function test parameters at his most recent assessment. The patient took Salbutamol 1.5 h prior to spirometry which may have improved results; however, pulmonary function parameters were significantly improved compared to prior tests and there was no significant post bronchodilator change. These factors in association with clinical control less than two weeks before his death, make intimacy-induced asthma less likely and anaphylaxis remains the suspected diagnosis.

With increasing popularity of relationship applications, especially popular amongst stigmatized populations, this case highlights the importance of allergy awareness as well as patient education and advocacy to decrease risk, particularly in the adolescent population, who are already at increased risk of severe anaphylaxis, especially in the context of asthma. Administration of epinephrine without delay can be life-saving [9]. This case highlights the importance of education around indications for epinephrine administration, proper technique, and prompt use in suspected anaphylaxis, particularly with immediate despite isolated respiratory or cardiovascular symptoms. Amongst those participating in intimate activities, disclosure of one’s allergies warrants discussion. This case is instructive: it is the first report of intimacy-related anaphylaxis among the men-who-have-sex-with-men population, the first involving an adolescent patient, the first case of food allergen transfer during insertive fellatio, and the first case of death from such an encounter. This case is novel and demonstrates the crucial need for increased advocacy in food allergy, education around intimacy-related anaphylaxis, and highlights the importance of allergy prevention across all populations.

## Data Availability

Not applicable.

## Abbreviations

IgE: Immunoglobulin E
MDI: Metered dose inhaler
SPT: Skin prick test
